# Effect of anatomical liver resection on early postoperative recurrence in patients with hepatocellular carcinoma assessed based on a nomogram: a single-center study in China

**DOI:** 10.3389/fonc.2024.1365286

**Published:** 2024-02-27

**Authors:** Ruizi Shi, Jianjun Wang, Xintao Zeng, Hua Luo, Xiongxin Yang, Yangjie Guo, Long Yi, Hong Deng, Pei Yang

**Affiliations:** Department of Hepatobiliary Surgery, Mianyang Central Hospital, School of Medicine, University of Electronic Science and Technology of China, Mianyang, China

**Keywords:** carcinoma, hepatocellular, hepatectomy, logistic models, alkaline phosphatase, postoperative complications

## Abstract

**Introduction:**

We aimed to investigate risk factors for early postoperative recurrence in patients with hepatocellular carcinoma (HCC) and determine the effect of surgical methods on early recurrence to facilitate predicting the risk of early postoperative recurrence in such patients and the selection of appropriate treatment methods.

**Methods:**

We retrospectively analyzed clinical data concerning 428 patients with HCC who had undergone radical surgery at Mianyang Central Hospital between January 2015 and August 2022. Relevant routine preoperative auxiliary examinations and regular postoperative telephone or outpatient follow-ups were performed to identify early postoperative recurrence. Risk factors were screened, and predictive models were constructed, including patients’ preoperative ancillary tests, intra- and postoperative complications, and pathology tests in relation to early recurrence. The risk of recurrence was estimated for each patient based on a prediction model, and patients were categorized into low- and high-risk recurrence groups. The effect of anatomical liver resection (AR) on early postoperative recurrence in patients with HCC in the two groups was assessed using survival analysis.

**Results:**

In total, 353 study patients were included. Multifactorial logistic regression analysis findings suggested that tumor diameter (≥5/<5 cm, odds ratio [OR] 2.357, 95% confidence interval [CI] 1.368–4.059; *P* = 0.002), alpha fetoprotein (≥400/<400 ng/L, OR 2.525, 95% CI 1.334–4.780; *P* = 0.004), tumor number (≥2/<2, OR 2.213, 95% CI 1.147–4.270; *P* = 0.018), microvascular invasion (positive/negative, OR 3.230, 95% CI 1.880–5.551; *P* < 0.001), vascular invasion (positive/negative, OR 4.472, 95% CI 1.395–14.332; *P* = 0.012), and alkaline phosphatase level (>125/≤125 U/L, OR 2.202, 95% CI 1.162–4.173; *P* = 0.016) were risk factors for early recurrence following radical HCC surgery. Model validation and evaluation showed that the area under the curve was 0.813. Hosmer-Lemeshow test results (*X* ^2^ = 1.225, *P* = 0.996 > 0.05), results from bootstrap self-replicated sampling of 1,000 samples, and decision curve analysis showed that the model also discriminated well, with potentially good clinical utility. Using this model, patients were stratified into low- and high-risk recurrence groups. One-year disease-free survival was compared between the two groups with different surgical approaches. Both groups benefited from AR in terms of prevention of early postoperative recurrence, with AR benefits being more pronounced and intraoperative bleeding less likely in the high-risk recurrence group.

**Discussion:**

With appropriate surgical techniques and with tumors being realistically amenable to R0 resection, AR is a potentially useful surgical procedure for preventing early recurrence after radical surgery in patients with HCC.

## 1 Introduction

Hepatocellular carcinoma (HCC), the most common type of primary liver cancer and the most common pathohistological type, has been reported to account for ~90% of cases (1). Currently, HCC treatment involves a multidisciplinary approach, multiple therapeutic approaches, and individualized treatment. Common treatments include surgery, ablation, transcatheter arterial chemoembolization, radiation therapy, and systemic antitumor therapy.

Patients with early HCC recurrence typically have a poorer prognosis than those with late recurrence. Kim et al. (2) noted that the risk rate of recurrence following HCC treatment peaked at one year (21.7%) and gradually declined after five years. Surgical resection modalities for HCC are divided into anatomical liver resection (AR) and non-anatomical liver resection (NAR), with NAR involving a local resection 1–2 cm from the tumor margins without regard to Couinaud liver segments, which can facilitate the preservation of hepatic parenchyma. AR was first proposed by Makuuchi et al. (3) as a systematic resection of liver segments limited by tumor portal vein branches to reduce the incidence of liver tumor cells invading the hepatic vascular system and transferring along the blood vessels. The effect of AR and NAR on the early prognosis of patients remains unclear, with some studies suggesting that AR is more likely to improve oncologic outcomes. In the retrospective study of 362 patients by Zhong et al., after propensity score matching (PSM), one-, three-, and five-year disease-free survival (DFS) rates were reported to be 51.1%, 44.7%, and 42.0%, respectively, in the AR group, and 44.9%, 34.3%, and 26.4%, respectively, in the NAR group (*P* = 0.039). Using multifactorial regression analysis, AR was reported to be an independent favorable prognostic factor in patients with HCC in combination with microvascular invasion (MVI) (HR 1.054, 95% CI 1.105–2.045, *P* = 0.009) (4). However, it has also been suggested that there is no significant difference in the effects of the two surgical procedures on patient DFS rates. In a retrospective study by Elvan et al., that included 94 samples (5), one-, three-, and five-year DFS rates in the NAR group were 73.6%, 39.1%, and 32.8%, respectively, and 48.8%, 22.7%, and 22.7%, respectively, in the AR group (*P* = 0.085). However, most of the above studies utilized a single baseline characteristic (for example, whether the tumor diameter exceeded 5 cm or whether MVI accompanied it) for survival analysis, with less consideration for each patient’s comprehensive situation. As postoperative recurrence in patients with HCC is a highly heterogeneous outcome involving the interaction of many potential factors, our stratified randomized study aimed to develop more comprehensive baseline characteristics using a self-developed recurrence prediction model to group our study population accordingly and to investigate the effect of each surgical modality on early recurrence of HCC.

## 2 Materials and methods

### 2.1 Study population and data sources

In this study, we selected 428 patients who underwent hepatectomy for primary hepatic malignancy at the Department of Hepatobiliary Surgery, Mianyang Central Hospital, between January 2015 and August 2022. In total, 353 patients with HCC (males, n = 289; females, n = 64; age range, 18–84 years) who met the inclusion criteria were enrolled. In accordance with guidelines for human subjects research, this retrospective study was approved by the institutional review board of the Institutional Ethics Review Board of Mianyang Central Hospital.

### 2.2 Selection criteria

Inclusion criteria comprised the following: (i) patients who had undergone surgical treatment and whose HCC diagnosis had been confirmed pathologically; (ii) patients who had not undergone preoperative transcatheter arterial chemoembolization (TACE), radiotherapy, or other treatments; (iii) patients with no distant metastases to the lungs and bones; and (iv) patients who had undergone >12 months follow-up post-treatment, and who had complete follow-up medical record data. Exclusion criteria comprised the following: (i) patients with non-primary HCC; (ii) patients with combined distant metastases; (iii) patients who had undergone preoperative TACE and radiotherapy; (iv) patients with a combination of other tumors or postoperative pathology suggestive of non-HCC; (v) patients lacking clinically important information; and (iv) patients who declined to attend follow-up or who were not included in follow-up post-discharge.

We initially screened 428 patients and excluded 13 patients with incomplete data or postoperative loss to follow-up, 17 patients who died postoperatively owing to non-tumor recurrence factors, 42 patients with postoperative pathology suggestive of hepatic cholangiocarcinoma or other types of tumors, and three patients with non-R0 resection.

### 2.3 Clinical features

We collected the following patient data: age, sex, Child-Pugh classification, presence of cirrhosis, and concomitant portal hypertension; serological parameters, namely, alpha-fetoprotein (AFP), alanine aminotransferase (ALT), aspartate aminotransferase (AST), γ-glutamyltransferase (γ-GT), total bilirubin (TBIL), neutrophil/lymphocyte ratio (NLR), alkaline phosphatase(ALP); viral infection data (hepatitis B virus [HBV]/hepatitis C virus [HCV]); surgery and postoperative recovery (AR or NAR), whether intraoperative blood transfusion had been performed, the amount of intraoperative bleeding, whether postoperative complications and hepatic failure occurred, and the duration of postoperative hospitalization; tumor condition and pathological features (tumor diameter, number of satellite nodules, and the presence of microvascular invasion (MVI); and prognosis (DFS at 12 months postoperatively in both groups).

All patients underwent a preoperative evaluation. Preoperative blood tests, liver and renal function, coagulation function, and other tests were performed to assess the patients’ general status, including preoperative with higher HBV-DNA levels and alanine aminotransferase (ALT) levels >2 times the upper limit of normal values. Antiviral and hepatoprotective treatments were first administered, and surgery was performed within a limited period once liver function had improved; liver function reserve was assessed using the Child-Pugh classification and the indocyanine green 15 min storage rate; preoperative computed tomography (CT) and magnetic resonance imaging (MRI) of the upper abdomen were performed to determine the tumor location and diameter; and three-dimensional reconstruction was used to measure the volume of the remaining liver and to determine the presence of vascular variation.

### 2.4 Principles of the surgical protocol

Surgery was performed by an authorized senior hepatobiliary surgeon who ensured there was no microscopic tumor residue at all surgical margins (R0 resection) and that the remaining liver volume was >40%. The surgeon developed an individualized hepatic resection surgical plan according to tumor location and diameter and determined whether there was vascular variation involvement.

### 2.5 Patient follow-up

All patients were followed postoperatively via telephone and outpatient visits. During the first six months postoperatively, liver function, alpha-fetoprotein (AFP), and abdominal ultrasonography examinations were performed monthly. These examinations were repeated every 3–6 months for 12 months, with a review undertaken every six months after that. Abdominal enhancement CT was repeated every six months postoperatively. If AFP levels were persistently elevated or recurrent metastasis was suspected on abdominal ultrasonography, further refinement of the abdominal enhancement CT or MRI was performed to evaluate disease recurrence, which was determined to be the time interval between the date of surgery and the date of diagnosis of tumor recurrence, the patients who relapse within 12 months after surgery are defined as early recurrence. All patients were followed up until April 2023.

### 2.6 Patient groups

An optimal cut-off value of 112 was calculated based on the principle of the maximum Youden index. Patients with scores higher than the optimal cut-off value were included in the high-risk group for early postoperative recurrence; those with scores lower than the optimal cut-off value were included in the low-risk group for early postoperative recurrence. The two groups of patients were divided into two subgroups based on the surgical modality (AR or NAR).

### 2.7 Statistical analysis

Statistical analyses were performed using SPSS version 26.0 software. Frequencies (constitutive ratios) were used to express count data, and measures were expressed as mean ± standard deviation ( $\overset{¯}{\text{x}}$ ± SD) if they conformed to normal distribution and median (interquartile spacing) for non-normal distribution. Count data were compared using a *chi*-squared test or an exact probability method. Measures that conformed to a normal distribution were compared using a *t*-test, and those that did not conform to a normal distribution were tested using a Mann–Whitney U test. All variables in this study were analyzed using logistic one-way regression; factors significantly associated with early recurrence were initially screened at *P* < 0.05, statistically significant factors were analyzed again using multifactorial analysis and considered statistically significant at *P* < 0.05, and nomograms were plotted using R4.2.0 software.

Using a receiver operating characteristics (ROC) curve, we determined the area under the curve of the predictive model, verified the differentiation of the model, maximized the Youden index to find the optimal cut-off value of the predictive model, calculated the sensitivity and specificity of the predictive model, and verified the accuracy of the model using self-sampling resampling with 1000 bootstrap iterations.

The recurrence index and the optimal cut-off value were calculated for each patient using the model score, and the patients were stratified into either low- or high-risk recurrence groups based on the score. We categorized patients into AR and NAR subgroups using surgical modality as an exposure measure, and the effects of different surgical modalities (AR or NAR) on the early recurrence of differing patients with HCC were determined using survival analysis.

### 2.8 Ethics approval

The studies involving human participants were reviewed and approved by the Institutional Ethics Review Board of Mianyang Central Hospital (Approval No. S20230352-02). Informed consent was obtained from all the patients for their data to be used for research purposes.

## 3 Results

### 3.1 Patient characteristics

In accordance with our inclusion and exclusion criteria, 17 patients with non-recurrence-related deaths were excluded (comprising six deaths during hospitalization; of these six patients, three with AR, the other three with NAR, and 11 deaths from other causes post-discharge). Thirteen patients were lost to follow-up post-discharge, and 42 patients had postoperative pathology suggesting cholangiocarcinoma or tumors of other origins (including 38 tumors of bile duct origin and four tumors of either rectal, gallbladder, breast, or other origins). In total, 353 patients who had undergone radical HCC resection were included for analysis in this study. There were 115 (32.5%) patients with early recurrence within 12 months postoperatively and 238 (67.5%) without recurrence at the end of 12-month follow-up.

We analyzed 353 patients using logistic one-way regression analysis. Eleven risk factors with *P-*values <0.05 were screened out as not being statistically significantly associated with early postoperative recurrence. Thirteen risk factors associated with early recurrence were screened (**Table 1**). Further logistic multifactorial regression analyses were performed using these thirteen risk factors. A logistic regression model was constructed using the aforementioned six factors as independent variables (**Table 2**).

**Table 1:** Clinicopathologic features of 353 patients.

Clinicopathologic features	Total population (n=353)	*B*	*P*	Exp(B)	95%*CI* for Exp(B)
Gender (Male/Female)	289 (81.9%)/64 (18.1%)	0.074	0.802		
Age (year)	54.59 ± 10.89	-0.005	0.634		
Days of hospitalization (day)	19 (14, 25)	0.021	0.106		
Operation time (min)	240 (172, 310)	<0.001	0.680		
Postoperative complications (Occurred/Not occurred)	27 (7.6%)/326 (92.4%)	0.384	0.349		
Acute liver failure (Occurred/Not occurred)	6 (1.7%)/347 (98.3%)	0.035	0.968		
Satellite nodule (Positive/Negative)	40 (11.3%)/313 (88.7%)	1.542	<0.001	4.674	2.334-9.362
AFP (≥400ng/ml/<400ng/ml)	66 (18.7%)/287 (81.3%)	1.309	<0.001	3.701	2.128-6.438
TBIL	14.6 (10.89, 19.5)	-1.371	0.752		
Virus (HBV/HCV) (Positive/Negative)	308 (87.3%)/45 (12.7%)	1.075	0.012	2.931	1.266-6.786
Cirrhosis (Positive/Negative)	276 (78.2%)/77 (21.8%)	0.664	0.028	1.943	1.075-3.513
Portal hypertension (Positive/Negative)	104 (29.5%)/249 (70.5%)	0.312	0.203		
Intraoperative blood transfusion (Occurred/Not occurred)	34 (9.6%)/319 (90.4%)	0.134	0.722		
Intraoperative bleeding(ml)	200.0 (100.0, 500)	0.001	0.010	1.000	1.001
Tumer numbers (≥2/<2)	62 (17.6%)/291 (82.4%)	0.983	0.001	2.673	1.528-4.677
Tumor diameter (≥5cm/<5cm)	144 (40.8%)/209 (59.2%)	1.466	<0.001	4.286	2.700-6.949
Vascular invasion (Occurred/Not occurred)	26 (7.4%)/327 (92.6%)	2.627	<0.001	13.839	4.643-41.247
Resection mode (AR/NAR)	147 (41.6%)/206 (58.4%)	1.014	<0.001	2.757	1.744-4.358
MVI (Positive/Negative)	136 (38.5%)/217 (61.5%)	1.694	<0.001	5.441	3.361-8.806
γ-GT (>40U/L/≤40U/L)	234 (66.3%)/119 (33.7%)	0.590	0.020	1.803	1.098-2.961
NLR	2.619 (1.799, 3.639)	0.106	0.073		
ALP (>125 U/L/≤125 U/L)	65 (18.4%)/288 (81.6%)	1.031	<0.001	2.803	1.617-4.860
ALT (>40U/L/≤40U/L)	166 (47.0%)/187 (53.0%)	0.410	0.072		
AST (>40U/L/≤40U/L)	164 (46.5%)/189 (53.5%)	0.655	0.004	1.924	1.226-3.020
CNLC stage (I/II/III/IV)	166 (47.0%)/104 (29.5%)/41 (11.6%)/42 (11.9%)	1.071	<0.001	2.920	2.249-3.790

AFP, alpha-fetoprotein; TBIL, total bilirubin; HBV, hepatitis B virus; HCV, hepatitis C virus; NAR, non-anatomical liver resection; AR, anatomical liver resection; MVI, microvascular invasion; γ-GT, γ-glutamyltransferase; NLR, neutrophil/lymphocyte ratio; ALP, alkaline phosphatase; ALT, alanine aminotransferase; AST, aspartate aminotransferase; CNLC, China Liver Cancer.

**Table 2:** Further logistic multifactorial regression analyses.

Clinicopathologic features	*B*	*P*	Exp (B)	95%*CI* for Exp (B)
MVI (positive/negative)	1.173	<0.001	3.230	1.880-5.551
AFP (≥400/<400) (ng/L)	0.926	0.004	2.525	1.334-4.780
Tumer numbers (≥2/<2)	0.794	0.018	2.213	1.147-4.270
Tumor diameter (≥5/<5) (cm)	0.857	0.002	2.357	1.368-4.059
Vascular invasion (positive/negative)	1.498	0.012	4.472	1.395-14.332
ALP (>125/≤125) (U/L)	0.789	0.016	2.202	1.162-4.173

AFP, alpha-fetoprotein; MVI, microvascular invasion; ALP, alkaline phosphatase.

**Figure 1:**
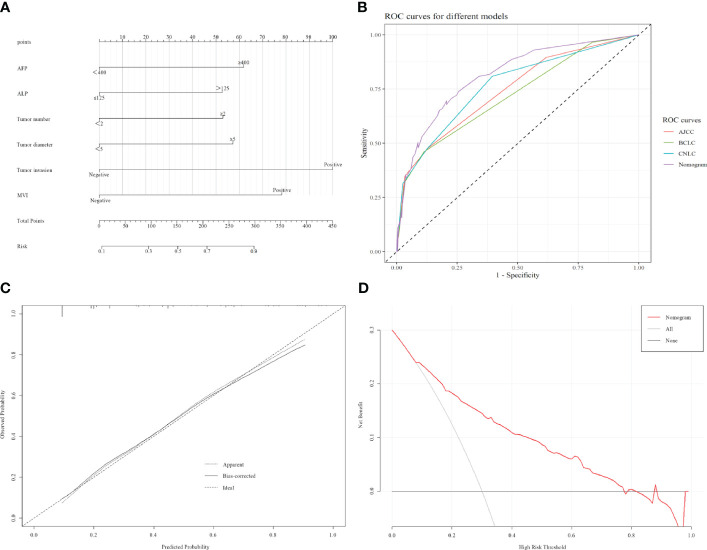
Construction and validation of the model. **(A)** Nomogram that can predict the risk of recurrence within 1 year. **(B)** ROC curve of nomogram, AJCC, BCLC, and CNLC staging systems. AJCC, American Joint Committee on Cancer, BCLC, Barcelona Clinic Liver Cancer, CNLC, China Liver Cancer. **(C)** Calibration curves of the radiomics nomogram. **(D)** Decision curve analyses for using the nomogram.

According to the constructed models, a predictive model was constructed for this modeling cohort (**Figure 1A**), and AJCC, BCLC, and CNLC staging systems were used for simultaneous comparisons using ROC analysis. Our findings concerning the modeling cohort were as follows: area under the ROC curve, 0.813 (95% CI, 0.7650.861, *P* < 0.001) and C-index, 0.813. The area under the ROC curve in the predicted model was greater than in the three staging systems, which indicated that the model was well discriminated (diagnostic sensitivity of the model, 69.6%; specificity, 79.4%) (**Figure 1B**). Model accuracy assessment and result visualization using the Hosmer-Lemeshow test and calibration curves are shown in (**Figure 1C**) (*χ^2^ = *1.255, *P* = 0.996, and *P* > 0.05), with calibration plots suggesting the predictive model had good accuracy. The model was also internally validated using bootstrap self-sampling with a repeat sample of 1000 cases and accurately distinguished postoperative patients who were likely to have early recurrence. With a threshold probability of approximately 0.07–0.78 using decision curve analysis (DCA) (**Figure 1D**), the decision curve showed that if the threshold probability of the patient is between 0.07 and 0.78, using nomogram model added more benefit than a treat-all-patients or treat-none scheme.

### 3.2 Population subgroups with HCC

**Figure 2:**
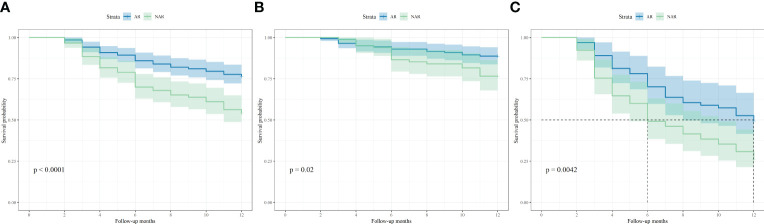
Kaplan-Meier curves of disease-free survival based on different surgical approaches in **(A)** all of 353 patients, **(B)** 224 patients in low-risk recurrence group and **(C)** 129 patients in high-risk recurrence group.

Recurrence scores were calculated separately for each patient based on predictive modeling, and an optimal cut-off value of 112 was obtained. The total population was stratified with patients having scores <112 categorized into a low-risk recurrence group and those having scores ≥112 stratified into a high-risk recurrence group (**Table 3**). A one-way logistic regression of risk factors was performed in both groups. In the low-risk recurrence group, the surgical resection method, tumor diameter, and MVI were found to be statistically significantly associated with recurrence within 12 months postoperatively. In the low-risk recurrence group, no macrovascular invasion was observed, whereas in the high-risk recurrence group, concomitant chronic viral hepatitis, vascular invasion, and a change in surgical procedure were important risk factors for early postoperative recurrence. We compared differences in the clinicopathologic features of the AR and NAR groups (**Table 4**). Differences in bilirubin levels (13.8 µmol/L vs. 15.7 µmol/L, respectively; *P* = 0.026) and alkaline phosphatase (7.7% vs. 17.1%, respectively; *P* = 0.033) were found to be statistically significant in patients in the low-risk recurrence group who underwent the two surgical procedures separately, while no differences were found in the clinicopathologic characteristics of the patients in the high-risk recurrence group. No differences were observed in the clinicopathological characteristics of patients in the high-risk recurrence group; however, differences in operative time, average hospitalization time, intraoperative bleeding, postoperative occurrence of liver failure, and related complications between the two surgical modalities were not statistically significant in patients in the low-risk recurrence group. In the high-risk recurrence group, patients in the AR group bled less than those in the NAR group (200 mL vs. 400 mL, respectively; *P* = 0.008) (**Table 5**). Through calculating survival outcomes in relation to AR versus NAR in both groups (**Figure 2**), both patient groups were found to benefit from AR for the prevention of early postoperative recurrence, with greater benefit of AR in patients in the high-risk recurrence group. DFS at four, eight, and twelve months in the low-risk recurrence group was 95.1%, 91.5%, and 88.7% in the AR group, respectively, and 95.1%, 84.0%, and 76.6% in the NAR group, *P* = 0.020, respectively. In the high-risk recurrence group, it was 81.3%, 60.5%, and 49.4% in the AR group at four, eight, and 12 months, respectively. DFS at four, eight, and twelve months in the NAR group was 64.6%, 41.5%, and 26.2%, respectively (*P* = 0.004).

**Table 3:** Relationship between clinical factors and early recurrence of low-risk recurrence group & high-risk recurrence group.

Clinical features	Low-risk recurrence group		High-risk recurrence group	
	HR	*P*	HR	*P*
Gender (Male/Female)	1.412 (0.512-3.892)	0.505	0.846 (0.329-2.171)	0.727
Age (year)	0.993 (0.960-1.028)	0.693	1.003 (0.973-1.035)	0.833
Days of hospitalization (day)	1.020 (0.982-1.060)	0.301	0.991 (0.952-1.032)	0.669
Operation time (min)	0.998 (0.994-1.001)	0.243	1.000 (0.996-1.004)	0.991
Postoperative complications (Occurred/Not occurred)	0.758 (0.164-3.490)	0.722	2.979 (0.616-14.403)	0.175
Acute liver failure (Occurred/Not occurred)	5.529 (0.338-90.545)	0.231	0.194 (0.020-1.921)	0.161
Satellite nodule (Positive/Negative)	3.450 (0.786-15.152)	0.101	1.793 (0.751-4.282)	0.188
AFP (≥400/<400) (ng/L)	0.526 (0.605-4.248)	0.547	1.482 (0.715-3.070)	0.290
TBIL (μmol/L)	1.104 (0.996-1.033)	0.131	0.990 (0.974-1.006)	0.226
Virus (HBV/HCV) (Positive/Negative)	1.022 (0.393-2.659)	0.964	8.977 (1.016-79.293)	0.048
Cirrhosis (Positive/Negative)	1.216 (0.536-2.761)	0.640	0.800 (0.228-2.811)	0.728
Portal hypertension (Positive/Negative)	0.937 (0.411-2.134)	0.876	1.500 (0.696-3.231)	0.300
Intraoperative blood transfusion (Occurred/Not occurred)	0.891 (0.248-3.201)	0.859	1.426 (0.415-4.906)	0.573
Intraoperative bleeding (ml)	1.000 (0.999-1.001)	0.614	1.000 (1.000-1.001)	0.166
Tumer numbers (≥2/<2)	1.583 (0.546-4.589)	0.397	1.571 (0.706-3.493)	0.268
Tumor diameter (≥5/<5) (cm)	2.249 (1.002-5.048)	0.049	1.073 (0.455-2.532)	0.873
Vascular invasion (Positive/Negative)	NA	NA	4.267 (1.373-13.266)	0.012
Resection mode (AR/NAR)	0.421 (0.203-0.874)	0.020	2.824 (1.349-5.912)	0.006
MVI (Positive/Negative)	2.504 (1.003-6.251)	0.049	1.006 (0.384-2.633)	0.991
γ-GT (>40/≤40) (U/L)	0.917 (0.442-1.901)	0.815	1.702 (0.722-4.012)	0.224
NLR	1.050 (0.884-1.247)	0.577	1.119 (0.905-1.383)	1.119
ALP (>125/≤125) (U/L)	2.375 (0.909-6.204)	0.077	1.411 (0.643-3.094)	0.391
ALT (>40/≤40) (U/L)	1.939 (0.935-4.023)	0.075	0.901 (0.441-1.839)	0.774
AST (>40/≤40) (U/L)	1.335 (0.642-2.776)	0.440	1.114 (0.535-2.319)	0.773
CNLC stage	1.896 (1.225-2.935)	0.004	2.295 (1.518-3.469)	<0.001

AFP, alpha-fetoprotein; TBIL, total bilirubin; HBV, hepatitis B virus; HCV, hepatitis C virus; NAR, non-anatomical liver resection; AR, anatomical liver resection; MVI, microvascular invasion; γ-GT, γ-glutamyltransferase; NLR, neutrophil/lymphocyte ratio; ALP, alkaline phosphatase; ALT, alanine aminotransferase; AST, aspartate aminotransferase; CNLC, China Liver Cancer.

NA, not applicable.

**Table 4:** Differences in the clinicopathologic features of the AR and NAR groups.

Clinical features	Low-risk recurrence group							High-risk recurrence group				
	AR	NAR		*t*/χ^2^/*Z*		*P*		AR	NAR	*t*/χ^2^/*Z*		*P*
Gender				0.519		0.471				1.419		0.234
Male	114	69						50	56			
Female	28	13						14	9			
Age (year)	55.69 ± 10.49	53.64 ± 10.54		-1.403		0.162		52.63 ± 12.07	55.31 ± 10.86	1.329		0.186
Satellite nodule				0.182		0.669				1.375		0.241
Positive	138	78						13	19			
Negative	4	4						51	46			
AFP				0.960		0.327				0.064		0.800
≥400ng/ml	9	2						36	38			
<400ng/ml	133	80						36	38			
TBIL (μmol/L)	13.8 (10.2,18.2)	15.7 (1.25,19.6)		-2.219		0.026		14.6 (11.0,20.4)	14.2 (11.2,20.1)	-0.506		0.613
Virus (HBV/HCV)				0.218		0.640				<0.001		1.000
Positive	116	69						61	62			
Negative	26	13						3	3			
Cirrhosis				0.059		0.808				0.110		0.704
Positive	100	59						57	60			
Negative	42	33						7	5			
Portal hypertension				3.574		0.059				0.189		0.664
Positive	32	28						23	21			
Negative	110	54						41	44			
Tumer numbers				0.521		0.470				0.062		0.803
≥2	13	10						20	19			
<2	129	72						44	46			
Tumor diameter				0.376		0.540				3.080		0.079
≥5cm	29	14						46	55			
<5cm	113	68						18	10			
Vascular invasion	N/A									1.620		0.203
Positive								10	16			
Negative								54	49			
MVI				1.330		0.249				0.569		0.451
Positive	15	13						52	56			
Negative	127	69						12	9			
γ-GT				0.428		0.513				1.271		0.260
>40U/L	86	46						48	54			
≤40U/L	56	36						16	11			
NLR	2.481 (1.622,3.390)	2.485 (1.701,3.463)		-0.361		0.718		2.711 (1.985,3.911)	3.000 (2.009,4.9268)	-0.751		0.452
ALP				4.560		0.033				0.103		0.748
>125 U/L	11	14						19	21			
≤125 U/L	131	68						45	44			
ALT				1.580		0.209				2.233		0.135
>40 U/L	57	40						30	39			
≤40 U/L	85	42						34	26			
AST				1.078		0.299				0.088		0.767
>40 U/L	49	34						41	40			
≤40 U/L	93	48						23	25			
CNLC stage				5.040		0.152				10.998		0.012
Stage I	92	60						6	8			
Stage II	38	12						32	22			
Stage III	10	8						15	8			
Stage IV	2	2						11	27			

AFP, alpha-fetoprotein; TBIL, total bilirubin; HBV, hepatitis B virus; HCV, hepatitis C virus; NAR, non-anatomical liver resection; AR, anatomical liver resection; MVI, microvascular invasion; γ-GT, γ-glutamyltransferase; NLR, neutrophil/lymphocyte ratio; ALP, alkaline phosphatase; ALT, alanine aminotransferase; AST, aspartate aminotransferase; CNLC, China Liver Cancer.

NA, not applicable.

**Table 5:** The effect of two surgical modalities in low-risk and high-risk recurrence groups.

Perioperative period features	Low-risk recurrence group					High-risk recurrence group				
	AR		NAR	*t*/χ^2^/*Z*	*P*	AR	NAR	*t*/χ^2^/*Z*	*P*	
Acute liver failure				0.117	0.732			0.274	0.600	
Occurred	2		0			3	1			
Not occurred	140		82			61	64			
Intraoperative blood transfusion				1.211	0.271			1.301		0.254
Occurred	11		10			4	9			
Not occurred	131		72			60	56			
Intraoperative bleeding (ml)	200.0 (92.5,400.0)		250.0 (100.0,500.0)	-1.062	0.288	200.0 (77.5,500.0)	400.0 (200.0,800.0)	-2.671	0.008	
Days of hospitalization (day)	18.0 (14.0,23.0)		17.5 (13.0,24.0)	-0.768	0.442	19.5 (15.0,27.0)	22.0 (17.0,26.5)	-1.393	0.164	
Operation time (min)	230.0 (155.0,306.3)		210.0 (153.75,290.0)	-0.990	0.322	240.0 (192.5,327.5)	280.0 (210.0,335.0)	-1.053	0.292	
Postoperative complications				0.213	0.644			1.523	0.271	
Occurred	11		55			3	8			
Not occurred	133		77			61	57			

NAR, non-anatomical liver resection; AR, anatomical liver resection.

## 4 Discussion

Currently, HCC is mainly treated via surgical resection using a multidisciplinary team approach (MDT, medical oncology, interventional medicine, and gastroenterology) and supplemented with local therapy, hepatic arterial cannulation for continuous chemotherapy infusion, and adjuvant therapy according to each patient’s physical condition and tumor stage. With advances in surgical techniques and increased usage of large medical centers, combined with the oncological characteristics of HCC, the use of AR has gradually become more prominent in hepatectomy, with many studies reporting a better prognosis with AR than with NAR (6–8).

The tumor-node-metastasis (TNM) staging system has been widely used to predict the survival rate for patients with tumors; however, in recent years, There are already many studies that have predicted prognoses using prediction models with higher accuracy and precision than TNM staging (9–11). Our model showed better performance than the AJCC, BCLC, and CNLC staging systems (0.813, 0.721, 0.743, and 0.763, respectively*; P* < 0.001). This may be because, in addition to the inclusion of assessment factors (tumor diameter, tumor number, and combined vascular invasion) that are widely used in these staging systems, we included other clinicopathological patient features, such as combined MVI, elevated AFP, and elevated ALP, which have been shown to significantly affect the prognosis of patients with HCC (12–14).

Previous studies have shown that the morphological characteristics of primary tumors and the early recurrence of postoperative are closely related to long-term prognosis in patients with HCC. The risk factors for HCC recurrence are largely related to the morphological characteristics of a patient’s tumor, e.g., tumor diameter ≥5 cm and portal vein invasion are indicative of increased invasiveness of the tumor and a higher risk of recurrence (15, 16). Moreover, the occurrence of MVI is closely related to a patient’s tumor load, differentiation level, AFP level, and HBV activity and replication (17), while the presence of satellite nodules in patients with MVI is suggestive of tumor progression (18).

Serum biomarker testing is also often used to predict HCC recurrence. ALP is a common liver function test, and an elevated ALP level is often in response to liver injury. Elevated serum ALP can also be used to evaluate the prognosis of bone diseases such as osteosarcoma, as well as tumors accompanied by bone metastases (19). Similarly, our study found that elevated ALP levels were positively correlated with the risk of early postoperative recurrence in patients (HR 2.803, 95% CI 1.617–4.860), which may be attributed to higher ALP activity in the nucleus of cancer cells and to the dynamic changes that occur during the cell cycle, suggesting that ALP may contribute to tumor formation through altering cell cycle regulation and proliferation (20). ALP can also be elevated in a number of inflammation-related diseases (e.g., hepatitis, choledocholithiasis, cholangitis, and pancreatitis), and the inflammatory milieu plays an important role in driving the development and progression of cancer (21); therefore, elevated serum ALP levels may reflect the fact that patients with HCC have more severe inflammation with a poorer prognosis. Alpha-fetoprotein (AFP) is a conventional tumor marker for predicting HCC recurrence, but its sensitivity is relatively low. des-γ-carboxy-prothrombin (DCP) was recently reported to be superior to AFP in detecting HCC recurrence (22, 23). Furthermore, biomarkers such as circulating tumor DNA assays and circulating tumor cells have also been noted in the improvement of AFP prediction performance (24). However, research concerning biomarkers or combinations of biomarkers that are highly accurate and easy to detect is ongoing.

The recurrence pattern of HCC has been extensively studied and mostly involves intrahepatic recurrence at the hepatic end of the residual liver (90.1%), with most recurrences occurring within 1–2 years postoperatively (54.5%) (18, 53). In 1998, Ueda et al. used CT hepatic angiography to determine the hemodynamics of HCC during carcinogenesis (25) and confirmed, for the first time, the theory of tumor blood flow (TBF). The TBF drainage area is considered to be the peritumor area where tumor blood drains and may contain more micrometastases than other areas. Therefore, to completely resect the peritumor area, the optimal surgical area for HCC should be determined based on the patient’s tumor hemodynamics, that is, the TBF drainage area. Based on this understanding, in 2000, Sakon et al. proposed the concept of three modes of intrahepatic recurrence of HCC, namely, (i) local intrahepatic metastasis, that is, recurrence of HCC spreading directly to the periphery of the tumor through portal venous blood flow or venous drainage; (ii) systemic intrahepatic metastasis, that is, HCC recurrence due to circulating tumor cells (CTCs), and (iii) multicenter HCC recurrence due to HCC redevelopment (26). Based on recurrence patterns after TBF hepatectomy, AR can be used to appropriately resect the drainage area of the TBF and effectively avoid the recurrence of HCC intrahepatic metastasis.

AR was first proposed by Makuuchi et al. with the aim of completely resecting the tumor-carrying portal branches supplied by the portal vein and the branches of the hepatic artery, thereby improving the surgical prognosis of patients with HCC. Owing to HCC’s tendency to progress to intrahepatic vascular structures (27, 28), AR allows complete resection of the tumor, peritumoral liver tissue, and possible micrometastases around the tumor. This procedure is preferred by most surgeons. However, NAR is typically used for patients with HCC with relatively deteriorated liver function reserves because of chronic hepatitis or cirrhosis. However, when comparing AR and NAR, it is also necessary to consider the difficulty of performing both surgical procedures as well as postoperative differences in complications among patients, such as residual liver ischemia and liver failure. Previous studies have shown that AR has a longer operative time and a higher likelihood of postoperative residual hepatic ischemia than NAR and that residual hepatic ischemia is an independent risk factor for postoperative recurrence in patients (29). In recent years, the gap between AR and NAR in terms of operative time, intraoperative bleeding, and the occurrence of serious postoperative complications has narrowed with the advancement of intraoperative aids and surgical techniques (30). Our study shows that in the low-risk recurrence group, no statistically significant difference was observed between the two surgical techniques in terms of operation time, average hospitalization time, intraoperative bleeding, postoperative liver failure, and related complications. In the high-risk recurrence group, patients in the AR group had less bleeding than those in the NAR group (200 mL vs. 400 mL, respectively; *P* = 0.008). This may be because, as surgical techniques have developed, the operative times for AR and NAR have become lesser. However, AR following hepatic vascular anatomy is more effective in avoiding intraoperative bleeding. DFS was superior to NAR in the low-risk group for recurrence, and this advantage was more pronounced in the subgroup of high-risk patients.

Previous studies have shown that the effect of surgical resection modality on the prognosis of patients with HCC, especially those at a relatively high risk of recurrence (4, 5, 31), was not statistically significant, whereas our study found that patients in the high-risk recurrence group who underwent AR had a significantly better one-year RFS rate than those who underwent NAR. This could be because some high-risk patients have CTCs that have not begun to spread through the bloodstream, and AR resection may be able to remove some CTCs that have not yet spread via blood metastases. However, the clinical application of CTCs remains challenging. The most important difficulty is that the earlier the cancer stage is, the fewer the CTCs. Furthermore, there are conflicting definitions of CTCs (32). Therefore, for patients with late tumor staging, we consider that risks and benefits should be balanced with consideration of the possible risk of high CTCs, the difficulty of intraoperative resection, and postoperative hepatic function compensation, and that AR should be selected to treat patients with surgical indications.

This single-center retrospective study had some limitations. We intend to plan a large multicenter large-sample study to explore a preoperative CTC model and enhance the validity of the model through internal and external validation, which may allow for a more in-depth investigation of changes in relation to the benefits of varying surgical modalities for different patient populations.

## Data availability statement

The raw data supporting the conclusions of this article will be made available by the authors, without undue reservation.

## Ethics statement

The studies involving humans were approved by Biomedical ethics committee of Mianyang Centre Hospital. The studies were conducted in accordance with the local legislation and institutional requirements. The participants provided their written informed consent to participate in this study. Written informed consent was obtained from the individual(s) for the publication of any potentially identifiable images or data included in this article.

## Author contributions

RS: Data curation, Writing – original draft, Writing – review & editing. JW: Data curation, Writing – original draft, Writing – review & editing. XZ: Methodology, Supervision, Writing – review & editing. HL: Supervision, Writing – review & editing. XY: Data curation, Investigation, Writing – review & editing. YG: Data curation, Investigation, Writing – review & editing. LY: Data curation, Investigation, Writing – review & editing. HD: Data curation, Software, Writing – review & editing. PY: Supervision, Writing – review & editing.
